# Supplementary material for: Evaluation of the Effect of a New Skin Fixation Technique to Avoid Shrinkage of Skin Samples Obtained from Canine Cadavers
Source: Animals (Basel). 2024 Sep 26;14(19):2791. doi: 10.3390/ani14192791 (PMC11482562; doi:10.3390/ani14192791)
Supplement: Supplementary file 1 [file animals-14-02791-s001.zip › animals-3211261-Supplementary.pdf]

Supplementary Material

Table S1. Median (min - max) of the specimens' from the thoracic, flank and upper thigh regions in different time periods**.**

	Immediately after excision, mm		10 min after excision, mm		After 48h fixed in 10% formalin, mm	
	**Control**	**Study**	**Control**	**Study**	**Control**	**Study**
Radius T-C-D	26.5 (23.6 - 28.7)	27.0 (24.6 - 29.8)	26.5 (23.6 - 28.7)	27.0 (24.6 - 29.8)	23.9 [c] (21.1 - 29.2)	29.6 [d] (27.0 - 33.1)
Radius T-C-Ca	26.3 (21.0 - 32.8)	25.0 (22.0 - 26.7)	26.3 (21.0 - 32.8)	25.0 (22.0 - 26.7)	25.1 [c] (20.1 - 28.0)	31.1 [d] (28.0 - 33.8)
Radius T-C-V	26.3 (23.0 - 30.4)	28.0 (24.8 - 30.8)	26.3 [a] (23.0 - 30.4)	28.0 [b] (24.8 - 30.8)	24.0 [c] (21.2 - 27.7)	30.0 [d] (26.7 - 34.1)
Radius T-C-Cr	25.8 (23.0 - 29.9)	25.8 (20.0 - 28.0)	25.8 (23.0 - 29.9)	25.8 (20.0 - 28.0)	25.4 [c] (20.4 - 30.5)	30.5 [d] (28.0 - 33.5)
Radius F-C-D	26.3 (20.6 - 30.7)	26.6 (22.0 - 27.9)	25.3 (19.7 - 28.6)	24.8 (21.0 - 27.1)	24.1 [c] (18.6 - 29.0)	29.1 [d] (27.0 - 32.5)
Radius F-C-Ca	27.0 [c] (23.1 - 29.0)	24.0 [d] (19.4 - 26.1)	25.6 (22.0 - 28.0)	24.0 (18.6 - 27.0)	26.0 [c] (22.1 - 27.6)	29.7 [d] (27.7 - 33.0)
Radius F-C-V	27.0 (22.0 - 28.7)	27.4 (22.0 - 30.7)	26.0 (20.0 - 28.6)	24.9 (21.4 - 31.0)	24.2 [c] (19.0 - 28.3)	29.9 [d] (21.7 - 35.0)
Radius F-C-Cr	26.2 (24.7 - 28.2)	25.5 (21.9 - 29.0)	25.7 (20.9 - 28.1)	24.5 (19.1 - 28.0)	24.8 [c] (22.6 - 27.6)	31.8 [d] (29.9 - 33.5)
Radius UT-C-D	23.5 (22.0 - 29.0)	25.0 (22.3 - 28.1)	23.0 (19.7 - 29.0)	24.0 (18.3 - 26.8)	22.4 c (18.0 - 28.0)	30.0 d (28.5 - 33.0)
Radius UT-C-Ca	26.9 (23.2 - 30.6)	27.6 (22.7 - 29.4)	25.3 (21.7 - 27.7)	25.5 (19.6 - 29.0)	24.6 c (20.7 - 29.0)	29.8 d (28.0 - 33.5)
Radius UT-C-V	26.2 (22.0 - 28.5)	25.0 (22.8 - 28.0)	24.8 (21.2 - 30.0)	24.5 (22.0 - 27.1)	24.3 c (20.6 - 30.0)	31.3 d (28.5 - 33.3)
Radius UT-C-Cr	26.6 (23.2 - 30.0)	26.9 (22.4 - 32.0)	25.4 (21.3 - 26.9)	25.4 (21.9 - 32.0)	24.2 c (22.4 - 28.0)	30.9 d (27.5 - 34.0)

a,b - $p<0,05$; c,d - $p<0,01$ different letters indicate statistically significant differences between Control and Study groups.

Table S2. Mean skin shrinkage of the specimens' diameter in thoracic, flank and upper thigh regions in different time periods.

	Immediately after excision, %		10 min after excision, %		After 48h fixed in 10% formalin, %	
	Control	Study	Control	Study	Control	Study
Diameter T-DV	-11.30<br>(1.66)	-6.66<br>(1.29)	-14.62<br>(1.38)	-15.38<br>(3.40)	-18.09<br>(2.37)<br>**	-0.42<br>(0.73)<br>**
Diameter T-CrCa	-14.05<br>(2.67)	-18.25<br>(1.83)	-13.77<br>(2.68)	-18.68<br>(1.46)	-17.04<br>(2.91)<br>**	-0.85<br>(0.57)<br>**
Diameter F-DV	-13.69<br>(2.02)	-13.27<br>(2.00)	-15.81<br>(2.61)	-17.28<br>(1.86)	-19.52<br>(2.81)<br>**	-0.72<br>(0.52)<br>**
Diameter F-CrCa	-12.49<br>(1.29)	-20.12<br>(2.11)	-14.98<br>(1.55)	-18.61<br>(2.65)	-16.53<br>(1.63)<br>**	-0.49<br>0.72<br>**
Diameter UT-DV	-15.51<br>(1.53)	-15.98<br>(2.02)	-20.71<br>(1.61)	-19.70<br>(1.64)	-21.73<br>(2.72)<br>**	-0.67<br>(0.37)<br>**
Diameter UT-CrCa	-12.52<br>(1.50)	-11.59<br>(2.50)	-15.94<br>(1.36)	-15.21<br>(2.51)	-16.55<br>(2.00)<br>**	-0.67<br>(0.80)<br>**

Reliability of percentage difference between Control-Study (Student's t test) $P < 0.05$, $P < 0.05$ *, $P < 0.01$ **
